# Characteristics of Choanal Atresia in Patients With Congenital Anomalies: A Retrospective Study

**DOI:** 10.7759/cureus.28928

**Published:** 2022-09-08

**Authors:** Ammar Habibullah, Ahmed M Mogharbel, Alwaleed Alghamdi, Abdulelah Alhazmi, Talal Alkhatib, Faisal Zawawi

**Affiliations:** 1 Otolaryngology - Head and Neck Surgery, King Abdulaziz University, Jeddah, SAU

**Keywords:** outcome, pediatric, congenital anomalies, syndromes, choanal atresia

## Abstract

Introduction

Choanal atresia (CA) is an uncommon congenital anomaly. There are various syndromes that are associated with CA. The purpose of this study is to determine the differences in CA's presentation and outcome when associated with other congenital anomalies and syndromes.

Method

This is a retrospective review study of all children (18 years and younger) who underwent CA repair in a tertiary referral healthcare center from January 2005 to April 2022. Demographics, comorbidities, radiological testing, operative reports, and outpatient reports were collected. Success was determined as a child with bilateral patent choana that is able to breathe from both nostrils comfortably.

Result

Twenty-four patients met the criteria for inclusion in this study. Bilateral CA was present in 15 (62.5%) patients. Mixed CA was the most common variant. There were various congenital anomalies in association with CA patients who are yet to be classified into a syndrome. The most common congenital anomaly was cleft lip and palate. Bony and mixed types were significantly associated with non-syndromic patients (p<0.05). Twenty patients (83%) were diagnosed with CA at age of less than one year, and four patients were diagnosed after one year of age. There were 36 surgeries performed on 24 patients, of which 27 were endoscopic and nine were using Hugher dilator. The overall success rate for CA repair was 50%. The median number of revisions per patient was 0.5.

Conclusion

CA is a challenging anomaly to repair. There are various factors that influence the outcome of children with CA. Otolaryngologists should counsel the patient and their families regarding possible need for revision especially in those with other craniofacial anomalies.

## Introduction

Choanal atresia (CA) is an uncommon congenital anomaly with an estimated prevalence of one in 7000 live births [1]. CA occurs when there is failure of canalization of the posterior nasal passage [2,3]. This is thought to be due to the persistence of the buccopharyngeal membrane; however, the pathogenesis of CA remains uncertain [2,3]. Multiple risk factors have been associated with CA including teratogenic medications, chromosomal anomalies, and syndromes such as CHARGE syndrome and trisomy 21 [4,5]. CHARGE syndrome is an autosomal dominant genetic disorder that was first described by Hall and Hittner. The main manifestations are coloboma, choanal atresia, and abnormal semicircular canals; other criteria include orofacial clefts and tracheoesophageal fistula [6,7].

CA could be either unilateral or bilateral, and literature suggests that the bilaterality of the disease is more likely associated with other congenital anomalies whereas unilateral CA is often an isolated anomaly [8]; furthermore, children with bilateral CA tend to present with severe airway distress and cyanosis as they are obligate nasal breathers [9]. CA is either bony in nature or a mixture of both membranous and bony, with mixed being the most common [9,10]. Our main goal is to study the associated risk factors for this disease and to highlight the anomalies associated with this disease.

## Materials and methods

Study design and setting

This is a retrospective review study in which we collected information from patients who were diagnosed with choanal atresia (CA) and followed up in a tertiary academic referral center between 2005 and 2021. We included all patients diagnosed with CA at age of less than 18 years within our study period; any patients above 18 years or not diagnosed with choana atresia or diagnosed with CA with no intervention were excluded from our study.

Data collection

Data were collected and categorized into four domains: (1) patient demographics including the age, gender, and nationality of the patients; (2) characteristics of CA including the side and type and age at diagnosis; (3) syndromic characteristics such as the type of syndrome, if any, and other congenital anomalies; and (4) intervention data, including the type of intervention, date of intervention, stenting usage, and reoperation rate, which was defined as the need to go back to the operating room for any reason related to the CA including dilation and revision surgeries.

Data analysis

Categorical variables were expressed in the form of the number and percentage, and their groups were compared using Pearson's chi-square test with Fisher's exact test. Non-normally distributed data were reported using median and range. Correlation analysis was conducted using Spearman's rank correlation coefficient for non-parametric variables. The significance is established when the two-sided P-value is <0.05. Statistical analysis was performed using SPSS software version 26 for Windows (SPSS Inc., Chicago, IL, USA). Figures were renovated using GraphPad Prism software version 8 (GraphPad Software Inc., San Diego, CA).

Confidentiality and ethical approval

Ethical approval was obtained from the King Abdulaziz University Hospital Ethical Review Board (reference number: 341-21). Access to data was available only to the principal investigator. To ensure the privacy and confidentiality of participants, all identifying variables have been removed.

## Results

Baseline demographic characteristics

**Figure 1:**
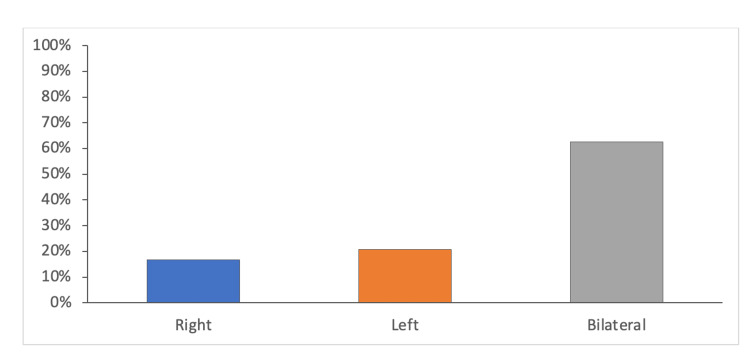
Bar graph highlighting the proportion of patients with unilateral (including side) and bilateral CA CA: choanal atresia

**Figure 2:**
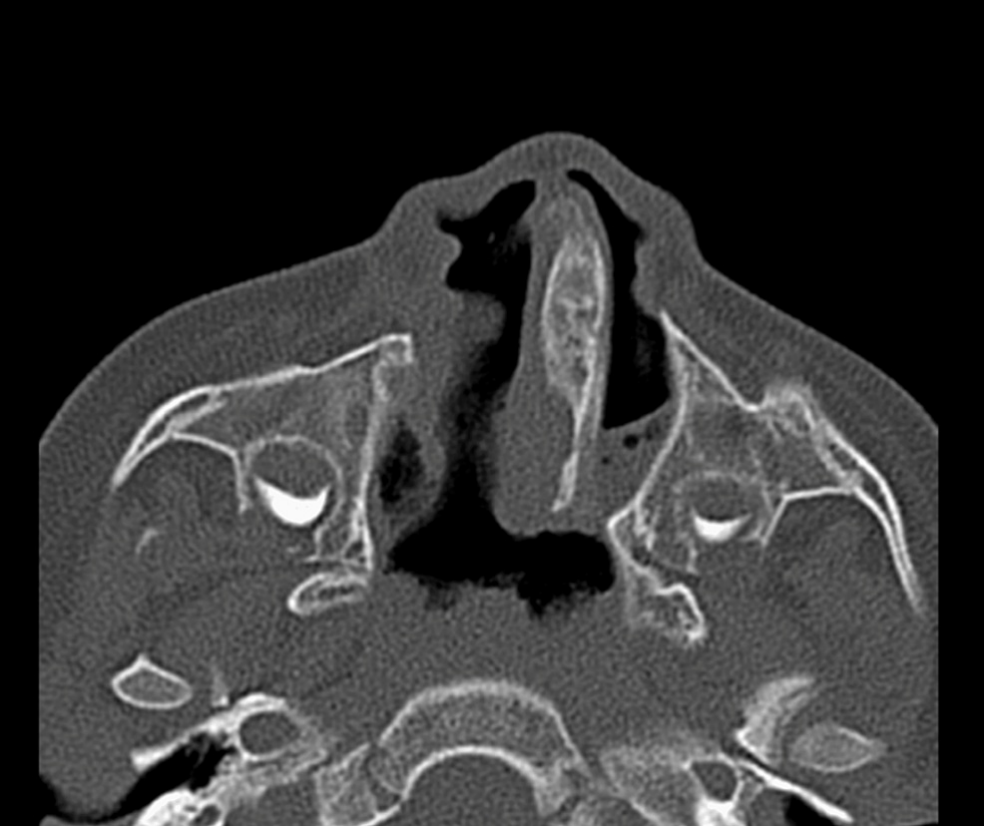
Unilateral choanal atresia This is an axial cut CT scan of a four-year-old male child showing left-sided mixed choanal atresia with a deviated nasal septum to the left side

**Figure 3:**
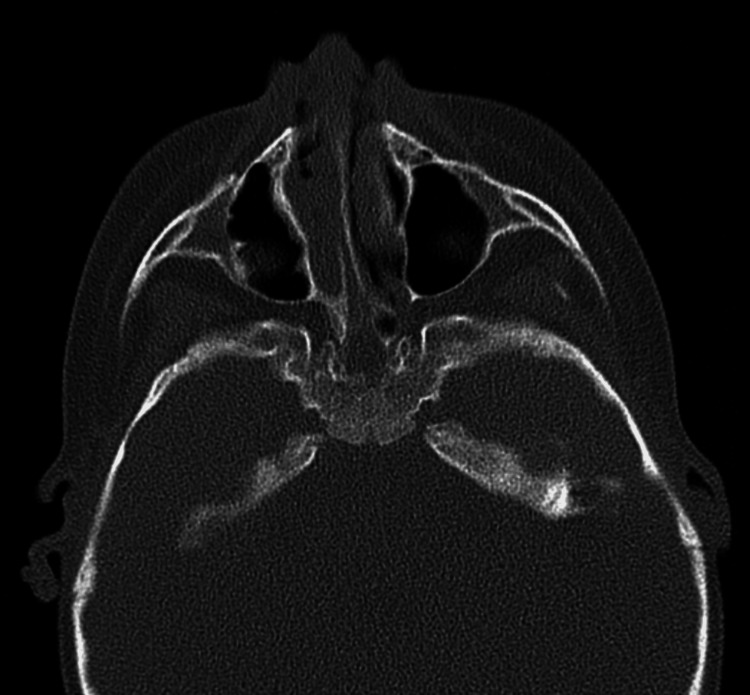
Bilateral choanal atresia This is an axial view CT scan of a one-month-old male infant showing right bony choanal atresia and left mixed choanal atresia

The present study included 24 patients with CA. The median age of the included patients was one (1-1800) day, in which 20 (83.33%) patients aged less than 12 months. Most of the included patients had non-syndromic choanal atresia: 20 (83.33%) patients. Bilateral choanal atresia was diagnosed among 15 (62.5%) patients, while left-side choanal atresia was confirmed among five (20.8%) patients (Table 1). Furthermore, 19 (79.2%) had mixed type, and bony choanal atresia was present in five (20.8%) (Figures 1, 2, 3).

**Table 1:** Baseline demographic characteristics of the included patients

Variables	Number	Percentage (%)
Age at diagnosis (days)	1 (1-1800)	
>12 months	4	16.66
<12 months	20	83.33
Associated syndromes		
Crouzons	1	4.2
Treacher-Collins	1	4.2
Trisomy 21	1	4.2
Alfie	1	4.2
Non-syndromic choanal atresia (CA)	20	83.33
Side of choanal atresia		
Right	4	16.7
Left	5	20.8
Bilateral	15	62.5
Type of choanal atresia		
Bony	5	20.8
Mixed	19	79.2
Other anomalies		
Yes	13	55.2
No	11	44.8

Out of the included patients, three (12.5%) had both cleft lip and cleft palate, while isolated cleft lip was documented among two (8.3%) patients. Two (8.3%) patients had ear anomalies, while one (4.2%) patient had congenital cataract. Nose anomalies were diagnosed among two (8.3%) patients, whereas hypothyroidism was diagnosed in one (4.2%) patient (Table 2).

**Table 2:** Congenital anomalies associated with choanal atresia RT: right; LT: left

Variables	Number	Percentage (%)
Cleft lip	2	8.3
Cleft lip and palate	3	12.5
Laryngeal cleft	1	4.2
Malformation of the larynx	1	4.2
Congenital subglottic stenosis	2	8.3
Nose anomalies (deviated nasal septum and nasal polyps)	2	8.3
Undescended testicle	1	4.2
Vocal cord paralysis	1	4.2
Hypothyroidism	1	4.2
Craniofacial dysostosis	1	4.2
Incomplete brain maturation	1	4.2
Ear anomalies (low-set ear and rocker bottom feet, small RT ear canal, and obliterated LT ear)	2	8.3
Rhinolith and adenoids	1	4.2
Aortic root dilation	1	4.2
Congenital cataract	1	4.2

There were two (8.33%) patients with right bony choanal atresia. Seven (29.16%) patients with bilateral CA had a mixed type. Of the included patients, 10 (41.66%) aged ≤12 months had mixed CA, while two (8.33%) patients aged >12 months had bony CA. There were six (25%) patients with congenital anomalies that had mixed-type CA (Table 3).

**Table 3:** Relation between type and other factors

Variables	Bony	Membranous	Mixed	P-value
	Number (%)	Number (%)	Number (%)	
Side of choanal atresia				
Right	2 (8.33)	1 (4.1)	1 (4.1)	0.489
Left	0 (0)	2 (8.33)	3 (12.5)	
Bilateral	3 (12.5)	5 (20.83)	7 (29.16)	
Age at diagnosis				
≤12 months	3 (12.5)	7 (29.16)	10 (41.66)	0.284
>12 months	2 (8.33)	1 (4.1)	1 (4.1)	
Syndromic choanal atresia				
No	3 (12.5)	6 (25)	11 (45.83)	0.136
Yes	2 (8.33)	2 (8.33)	0 (0)	
Congenital anomalies				
No	2 (8.33)	4 (16.66)	5 (20.83)	0.93
Yes	3 (12.5)	4 (16.66)	6 (25)	

Surgical interventions for choanal atresia

Endoscopic repair of choanal atresia was performed in 19 (79.2%) patients. Mitomycin was used in three (12.5%) patients, and a stent was inserted in 13 (54.16%). The recurrence of choanal atresia was confirmed among 12 (50%) patients. Subsequently, nine (37.5%) patients who developed recurrence of CA had bilateral CA preoperatively. In addition, 10 (14.66%) patients subjected to endoscopic surgery developed recurrence of CA, while seven (29.16%) patients treated with stent insertion developed recurrence of CA. One patient with trisomy 21 developed recurrence of CA, while no patient with Crouzons or Treacher-Collins syndromes developed recurrence of CA. Furthermore, six (25%) patients with congenital anomalies developed recurrence of CA (Tables 4, 5).

**Table 4:** Interventions for choanal atresia

Variables	Number	Percentage (%)
Surgical intervention		
Hugher	5	20.8
Endoscopic	19	79.2
Mitomycin C use	3	12.5
Stent insertion	13	54.16
Recurrence	12	50

**Table 5:** Recurrence of choanal atresia with different variables

Variables	Recurrence of choanal atresia		P-value
	No	Yes	
Side of choanal atresia			
Right	3 (12.5%)	1 (4.16%)	0.40
Left	3 (12.5%)	2 (8.33%)	
Bilateral	6 (25%)	9 (37.5%)	
Type of choanal atresia			
Bony	3 (12.5%)	2 (8.33%)	0.67
Mixed	9 (47.4%)	10 (52.6%)	
Surgical intervention			
Hugher	3 (12.5%)	2 (8.33%)	0.5
Endoscopic	9 (37.5%)	10 (14.66%)	
Use of mitomycin C			
Yes	1 (4.16%)	2 (8.33%)	0.5
No	11 (45.83%)	10 (41.6%)	
Use of stent			
Yes	6 (25%)	7 (29.16%)	0.5
No	6 (25%)	5 (20.83%)	
Syndromes			
No syndromes	10 (41.66%)	10 (41.66%)	1
Yes	2 (8.33%)	2 (8.33%)	
Crouzons	1 (4.16%)	0 (0%)	0.4
Treacher-Collins	1 (4.16%)	0 (0%)	
Trisomy 21	0 (0%)	1 (4.16%)	
Alfie	0 (0%)	1 (4.16%)	
Congenital anomalies			
Yes	7 (29.16%)	6 (25%)	0.682
No	5 (20.83%)	6 (25%)	

There were three (12.5%) patients with syndromic CA that had bilateral CA (Table 6). While seven (29.16%) patients with congenital anomalies had bilateral CA, four (16.66%) patients had left-sided CA. Endoscopic repair was performed among 13 (54.16%) patients with bilateral CA. Two (8.33%) patients with left-sided CA were treated with mitomycin C. Out of the included patients, 15 (62.5%) aged ≤12 months were subjected to endoscopic repair of CA, in contrast to four (16.66%) aged >12 months (Tables 7-9). Out of the included patients, two (8.33%) patients with left-sided CA received mitomycin C; whereas two (8.33%) patients aged ≤12 months received mitomycin C, one (4.1%) patient aged >12 months received it (P=0.437). Three (12.5%) patients with congenital anomalies were treated with mitomycin (Table 6).

**Table 6:** Associated factors This table highlights the associated factors in bilaterality and outcomes

Variables	Right	Left	Bilateral	P-value
	Number (%)	Number (%)	Number (%)	
Syndromic choanal atresia				
No	3 (12.5)	5 (20.83)	12 (50)	0.517
Yes	1 (4.1)	0 (0)	3 (12.5)	
Congenital anomalies				
No	2 (8.33)	1 (4.1)	8 (33.33)	0.425
Yes	2 (8.33)	4 (16.66)	7 (29.16)	
Intervention				
Hugher	2 (8.33)	1 (4.1)	2 (8.33)	0.276
Endoscopic	2 (8.33)	4 (16.66)	13 (54.16)	
With mitomycin	0 (0)	2 (8.33)	1 (4.1)	

**Table 7:** Association of bilaterality and co-anomalies and recurrence rate

Variables	Unilateral	Bilateral	P-value
	Number (%)	Number (%)	
Syndromic			
No	8 (33.33)	12 (50)	0.514
Yes	1 (4.1)	3 (12.5)	
Recurrence			
No	6 (25)	6 (25)	0.2
Yes	3 (12.5)	9 (37.5)	
Congenital anomalies			
No	3 (12.5)	8 (33.33)	0.3
Yes	6 (25)	7 (29.16)	

**Table 8:** Association of intervention and co-factors

Variables	Hugher	Endoscopic	P-value
	Number (%)	Number (%)	
Side of choanal atresia			
Right	2 (8.33)	2 (8.33)	0.276
Left	1 (4.1)	4 (16.66)	
Bilateral	2 (8.33)	13 (52)	
Age at diagnosis			
≤12 months	5 (20.83)	15 (62.5)	0.365
>12 months	0 (0)	4 (16.66)	
Syndromic choanal atresia			
No	3 (12.5)	17 (70.83)	0.074
Yes	2 (8.33)	2 (8.33)	
Congenital anomalies			
No	3 (12.5)	10 (41.66)	0.585
Yes	2 (8.33)	9 (37.5)	

**Table 9:** Association of the use of mitomycin C with co-factors

Variables	Mitomycin C		P-value
	No	Yes	
Side of choanal atresia			
Right	4 (16.66%)	0 (0%)	0.106
Left	3 (12.5%)	2 (8.33%)	
Bilateral	14 (58.33%)	1 (4.1%)	
Age at diagnosis			
≤12 months	18 (75%)	2 (8.33%)	0.437
>12 months	3 (12.5%)	1 (4.1%)	
Syndromic choanal atresia			
No	17 (70.83%)	3 (12.5%)	0.563
Yes	4 (16.66%)	0 (0%)	
Congenital anomalies			
No	11 (45.83%)	0 (0%)	0.141
Yes	10 (14%)	3 (12.5%)	

Factors associated with recurrence of choanal atresia

There was a negative correlation between the patients' age (r=-0.255, P=0.229) and recurrence of CA. There was a positive correlation between the side of CA (r=0.272, P=0.198), syndromic choanal atresia (r=0.037, P=0.863), and recurrence. In this concern, the use of mitomycin showed a positive correlation (r=0.126, P=0.557) with the recurrence of CA. The presence of congenital anomalies (r=-0.083, P=0.69) and the use of stent (r=-0.084, P=0.69) were negatively associated with recurrence of CA (Table 10).

**Table 10:** Regression analysis highlighting the correlation between study parameters and recurrence of choanal atresia

Variables	Correlation coefficient	P-value
Age of the patients	-0.255	0.229
Side of choanal atresia		
Right versus left versus bilateral	0.272	0.198
Unilateral versus bilateral	0.486	0.57
Type of choanal atresia	-0.019	0.92
Syndromic choanal atresia	0.037	0.863
Hugher intervention	0.103	0.633
Use of mitomycin C	0.126	0.557
Use of stent	-0.084	0.698
Congenital anomalies	-0.083	0.69

There was a statistically significant negative correlation between the age of the patients and the bilaterality of CA (r=-0.792, P<0.001), representing bilateral CA being more frequently diagnosed earlier in the patient's life. In this respect, patients with non-syndromic CA were associated with high risk of mixed type of CA (r=-0.443, P=0.03).

## Discussion

This study found that majority of the population was diagnosed with bilateral CA and with mixed-type predominance. They were less frequently associated with syndromes; however, the majority had other congenital anomalies rather than isolated CA. The approximate incidence of CA is between 1:4000 and 1:10000 live births [6] and is more often unilateral [10,11]; however, most of our population had bilateral CA, which could be due to referral bias to high-complexity centers such as ours. The composition of CA was historically classified into purely membranous, purely osseous, or mixed osseous-membranous [11]. Recently, the existence of a purely mixed type has been less frequently reported [3,12]. In our study, most cases were mixed type, consistent with findings in literature [12].

Multiple syndromes and congenital anomalies are commonly found among CA patients, especially in those with bilateral disease [6]. Associated congenital anomalies were found in more than half of our sample, with cleft lip and palate being the most found; other anomalies of the airways, nose, and ear were also noted. This is of importance as thorough screening for other anomalies is important in patients with CA to diagnose and treat other associated anomalies.

In our sample, we had four patients diagnosed with syndromes, namely, Crouzons, trisomy 21, Alfie, and Treacher-Collins. These showed lower occurrence of associated syndromes than reported in literature [13].

The endoscopic technique (nasal or retropalatal), with or without powered instrumentation, offers excellent visualization with great ease in removing the bony choanae [9]. Endoscopic intervention has a success rate of 96.3% and 86.2% for unilateral and bilateral CA, respectively [14], and was used for most of our cases. The use of mitomycin C topically as an adjunct to surgically repairing choanal atresia may offer improved patency with a decreased need for stenting, dilatations, and revision surgery [15]. However, based on our results, using mitomycin C did not significantly affect recurrence rates.

Post-operative stenting has not been proven to increase the chances of surgical success [16], even though authors still recommend the placement of stents in high-risk cases such as in neonates and in cases of bilateral CA [17]. A recently published meta-analysis of 15 studies evaluating bilateral CA repair found similar surgical success rates for stented versus non-stented patients, with stent durations varying from days to months [16]. Similarly, we found that stenting did not reduce recurrence rates or improve surgical success rates significantly.

This study was limited by its retrospective nature and missing data as well as that it is a single-center study, which limits the generalizability of study findings and results in small sample sizes. Further studies could be conducted with larger sample sizes from multiple centers to improve study results.

## Conclusions

CA is a challenging anomaly to repair. There are various factors that influence the outcome of children with CA. Otolaryngologists should counsel the patient and their families regarding possible co-factors and co-anomalies as well as the need for revision surgeries especially in those with other craniofacial anomalies.
